# Design and evaluation of 16S rRNA sequence based oligonucleotide probes for the detection and quantification of *Comamonas testosteroni *in mixed microbial communities

**DOI:** 10.1186/1471-2180-6-54

**Published:** 2006-06-13

**Authors:** Stephan Bathe, Martina Hausner

**Affiliations:** 1 Institute of Water Quality Control and Waste Management, Technical University of Munich, Am Coulombwall, 85748 Garching, Germany; 2 Department of Civil and Environmental Engineering, Northwestern University, 2145 Sheridan Road, Evanston IL 60208-3109, USA; 3 Department of Biological Sciences, The University of Warwick, Coventry CV4 7AL, UK

## Abstract

**Background:**

The β-proteobacterial species *Comamonas testosteroni *is capable of biotransformation and also biodegradation of a range of chemical compounds and thus potentially useful in chemical manufacturing and bioremediation. The ability to detect and quantify members of this species in mixed microbial communities thus may be desirable.

**Results:**

We have designed an oligonucleotide probe for use in fluorescent in situ hybridization (FISH) and two pairs of PCR primers targeting a *C. testosteroni *subgroup. The FISH probe and one of the PCR primer pairs are suitable for quantification of *C. testosteroni *in mixed microbial communities using FISH followed by quantitative image analysis or real-time quantitative PCR, respectively. This has been shown by analysis of samples from an enrichment of activated sludge on testosterone resulting in an increase in abundance and finally isolation of *C. testosteroni*. Additionally, we have successfully used quantitative PCR to follow the *C. testosteroni *abundance during a laboratory scale wastewater bioaugmentation experiment.

**Conclusion:**

The oligonucleotides presented here provide a useful tool to study *C. testosteroni *population dynamics in mixed microbial communities.

## Background

*Comamonas testosteroni *is a ubiquitously occuring β-proteobacterial species which has been isolated from aquatic as well as terrestrial environments. It has been shown to be capable of transformations of steroid compounds [1-4] and also of degradation of aromatic hydrocarbons [5-10]. *C. testosteroni *is thus of interest for potential biotechnological applications such as chemical transformations in fine chemical manufacturing [11] and bioremediation processes [7,12], as indicated by laboratory results.

Due to the widespread environmental distribution and the potential relevance of *C. testosteroni *in biotransformation processes, it may be of interest to follow the population dynamics of this species over time in a mixed-species environment. Since culture-dependent methods are not sufficiently accurate to detect and quantify one particular species within a mixed bacterial community, methods targeting the 16S rRNA and/or its gene(s) need to be applied to such a problem, as has already been done in previous investigations [13-16]. Probes for species of the genera *Comamonas *and *Delftia *(COM1424 [17], and PPT [18]) as well as for species of the family *Comamonadaceae *(CTE [19]) have been described earlier, but might lack the desired specificity. The use of reporter genes such as *gfp *or *dsRed *is also possible, but only applicable to environments with intentionally introduced labelled strains and not to the investigation of uncharacterized environmental samples. Additionally, the introduction of reporter genes may impair the environmental fitness of the introduced strain [20], and variations in reporter expression might interfere with detection. The use of reporter gene carrying strains also means working with genetically modified organisms, which may not be possible in all cases.

The following study reports on the development of 16S rRNA sequence based oligonucleotide probes which are suitable for detection and quantification of *Comamonas testosteroni *in mixed cultures using PCR and/or fluorescent in situ hybridization (FISH).

## Results

### 16S rRNA sequence diversity of *Comamonas testosteroni*

**Figure 1:**
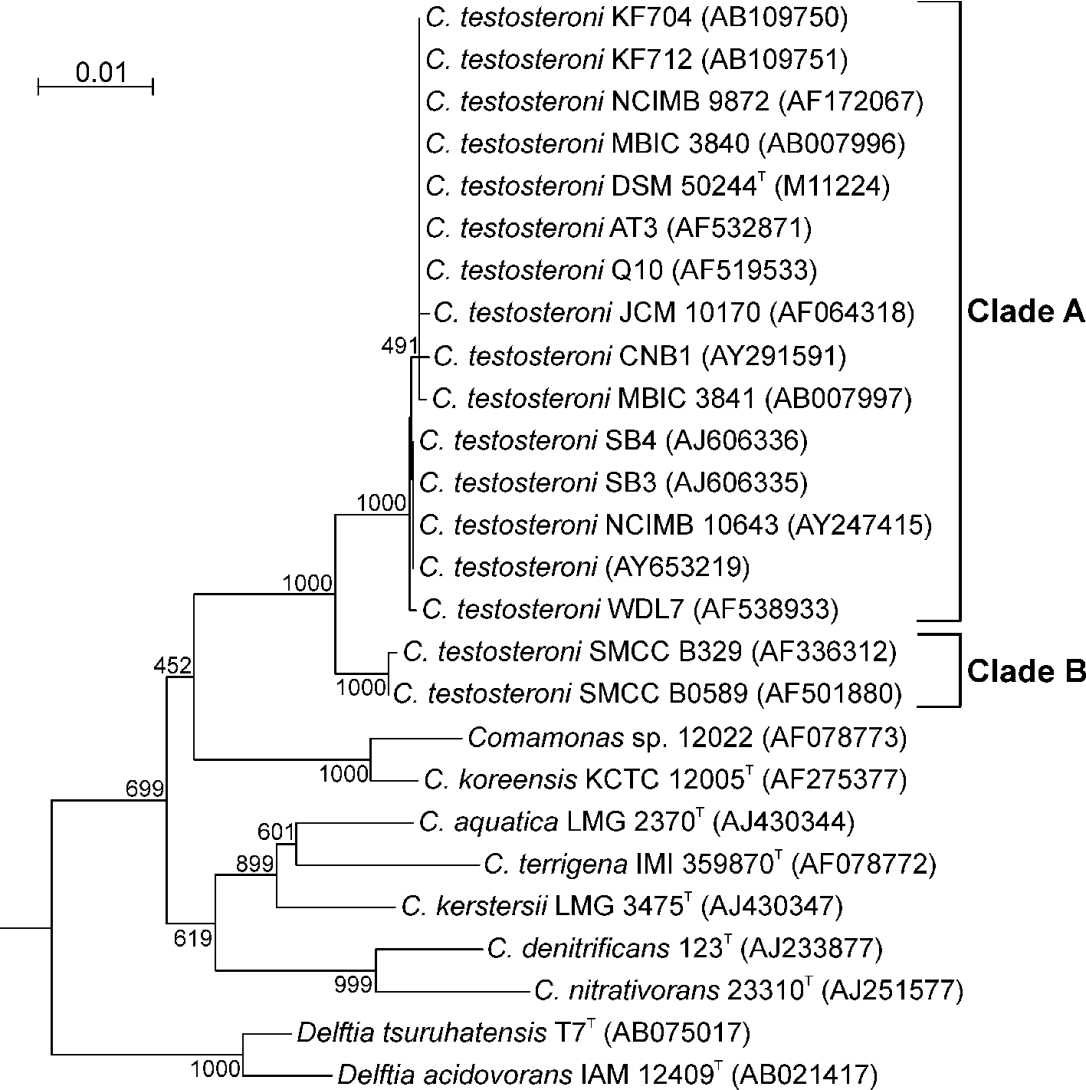
Neighbour-joining tree of 16S rRNA gene sequences of species of the genera *Comamonas *and *Delftia *with an emphasis on *Comamonas testosteroni *corresponding to *E. coli *positions 135-1369. The sequences of *Ideonella dechloratans *CCUG 30898^T ^[GenBank:X72724] and of *Rubrivivax gelatinosus *ATCC 17011^T ^[GenBank:D16213] were used as outgroups. Bootstrap values of 1000 resamplings are shown at the branch points.

A short survey of 16S rRNA sequence diversity of *C. testosteroni *was carried out. Fig. 1 shows a phylogenetic tree based on 16S rRNA sequences of species of the genera *Comamonas *and *Delftia*, with an emphasis on sequences of *C. testosteroni *isolates with more than 1200 bp length available from Ribosomal Database Project II. The *C. testosteroni *sequences form two clades (designated A and B) with a sequence divergence of approximately 1%. Clade A contains far more sequences than clade B, and all physiologically characterized strains as well as all strains isolated during studies dealing with biotransformation or biodegradation fall within this clade.

### Probe design

The sequence alignment from which the tree was constructed was then used as a basis to identify sequence regions which could be used for the design of FISH probes and PCR primers specific for *C. testosteroni*. Useful stretches were identified around nucleotides 450–480, 990–1020, and 1130–1160 (*E. coli *numbering). However, it was not possible to design oligonucleotides targeting simultaneously sequences from clades A and B together. Since clade A seemed to contain the organisms with a higher environmental and biotechnological relevance, we decided to design probes targeting this sequence cluster. Two PCR primer pairs and one FISH probe were designed (see Table 1). BLAST searches revealed that these oligonucleotides had a complete match only with sequences showing >99% identity to the sequence of the *C. testosteroni *type strain.

### Pure culture evaluation of the designed oligonucleotides

All oligonucleotides were evaluated in situ on a selection of proteobacterial pure cultures including all *C. testosteroni *strains available from the German Collection of Microorganisms (DSM). The tests revealed that the newly designed primers and probe were specific for the strains of this species. Primer pair CteA1 yielded a PCR product with a size of 571 bp, whereas primer pair CteA2 produced an amplicon of 167 bp length. It should be noted here that both primer pairs produced products from all examined *C. testosteroni *cultures, indicating that all tested cultures fell within clade A. A clade B culture was not available for testing, but since all developed oligonucleotides except primer CteA2-rev show 3 or more mismatches with the available clade B sequences, it can be expected that these strains would be excluded from detection by PCR or FISH. The CY3 labelled FISH probe CteA gave well-fluorescing signals when used with 30% of formamide in the hybridization buffer. Cells of all available *C. testosteroni *isolates except of strain DSM 6781 gave well-fluorescing signals when FISH was applied to paraformaldehyde fixed cells. To identify the reason for the reduced signal intensity in strain DSM 6781, its 16S rRNA sequence was determined [EMBL:AM113745]. The sequence had a central mismatch with probe CteA. Accordingly, we suggest to work with a degenerate version of the probe with the sequence CAT GAC CCG (G/A)GG ATA TTA GC. All non-target strains tested did not produce PCR products with primers CteA1 and CteA2, nor did they give a fluorescence signal when using probe CteA in FISH, although low background fluorescence was observed in some cultures.

### Analysis of testosterone enrichment cultures and isolates

In order to test the developed oligonucleotides on an unknown mixed culture, we provided conditions for the enrichment of *C. testosteroni *from activated sludge by two successive enrichment steps in liquid media containing testosterone as the sole source of carbon and energy and finally by spreading samples of the second culture on agar plates composed of the same medium. Samples of the activated sludge, the two enrichment cultures, and of five isolates obtained from the testosterone plates were then analysed by PCR and FISH.

**Figure 2:**
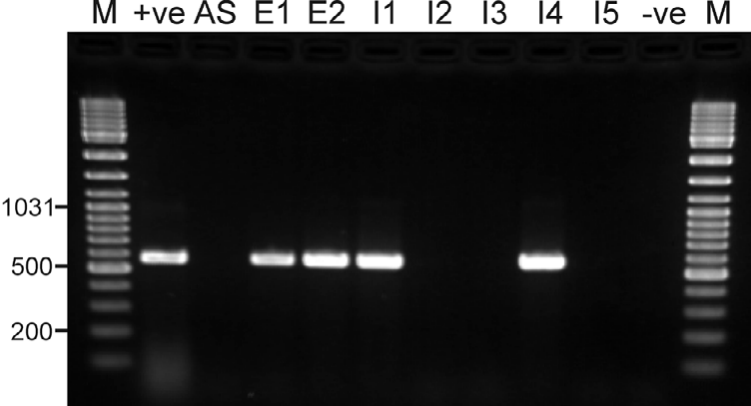
Agarose gel showing PCR products obtained using the primer pair CteA1 from the testosterone enrichment cultures and the isolates grown on testosterone plates. M: 100 bp ladder, +ve: positive control (*C. testosteroni *genomic DNA), AS: activated sludge inoculum, E1: first enrichment step, E2: second enrichment step, I1–I5: isolates, -ve: negative control (no template DNA).

Fig. 2 shows the results of an end-point PCR reaction (30 cycles) carried out using primer pair CteA1. No product was obtained from DNA from the activated sludge, suggesting a low initial abundance of *C. testosteroni*. In contrast, DNA samples from both enrichment cultures as well as two of the isolates produced a band at 570 bp, indicating a successful enrichment and isolation of *C. testosteroni*. When the PCR product amplified from the first enrichment culture was subjected to direct sequencing, a clean sequence [EMBL:AM113744] with 461 of the 462 obtained nucleotides identical to the sequence of *C. testosteroni *DSM 50244 was retrieved. Partial analysis of the 16S sequences of the isolates [EMBL:AM113739–AM113743] showed that the two isolates giving PCR products with the CteA1 primers displayed 100% sequence identity with the sequence of *C. testosteroni *DSM 50244, whereas the other isolates showed 100% identities to the sequences of *Pseudomonas *sp. B13 [GenBank:AF039489], *Pseudomonas *sp. WAS2 [GenBank:AB007999], and *Rhodococcus equi *[GenBank:AF490539], respectively. The two *C. testosteroni *strains could also be identified by FISH using the probe CteA.

In order to assess the suitability of the oligonucleotides for quantification of *C. testosteroni *in mixed cultures, primer pair CteA2 was used in real-time quantitative PCR and probe CteA was used for quantitative image analysis. The primer pair Eub341F/534R and the FISH probes Eub338 mix were used in parallel as a measure of total cell abundance. The ratios between *C. testosteroni *specific signals and the eubacterial specific signals will produce a fractional number representing the *C. testosteroni *abundance in a given sample. This approach has been applied to cells labelled by FISH [21] or fluorescent proteins [22] and has also been used in real-time PCR quantification [14].

**Figure 3:**
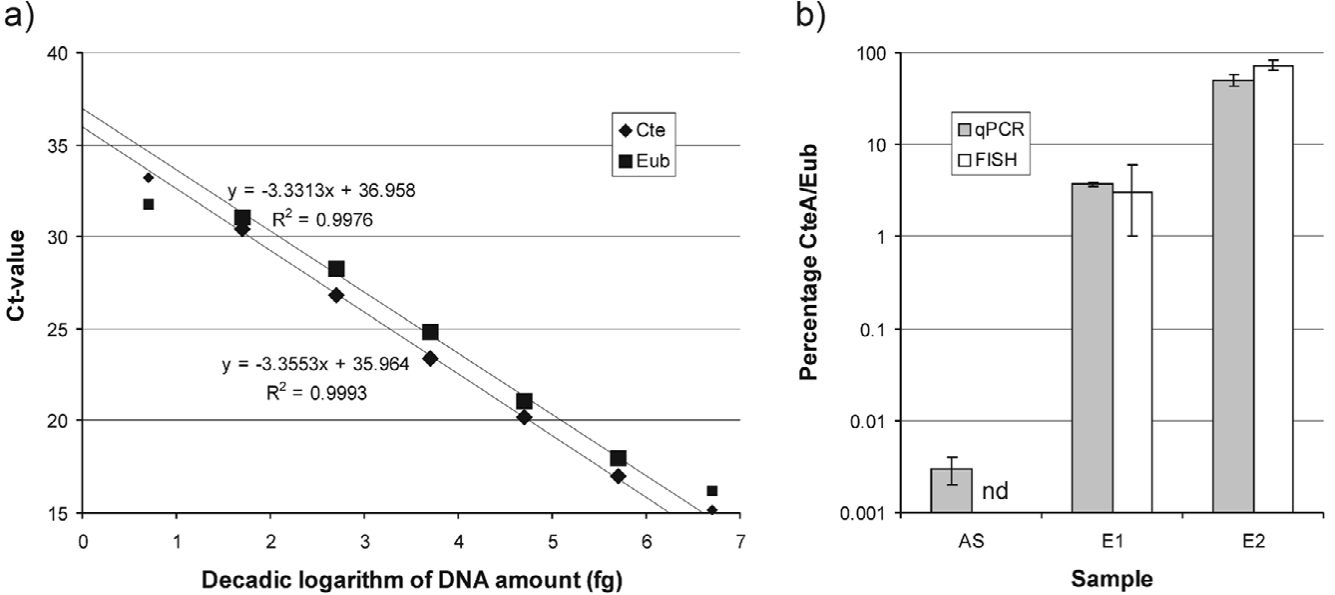
a) Real-time PCR standard curves obtained using primer pairs CteA2 (Cte, diamonds) and 341F/534R (Eub, squares) using 10fold serial dilutions of *C. testosteroni *genomic DNA as template DNA. Linear regressions are calculated using the large dots of each series. Error margins are too small to be visible. b) Abundances of *C. testosteroni *in the activated sludge inoculum (AS) and the two testosterone enrichment steps (E1 and E2) as measured by real-time PCR and quantitative FISH. nd: not detectable.

A dilution series of genomic DNA of *C. testosteroni *DSM 50244 yielded products from 5 fg on, which can be considered the detection limit, and showed a linear relationship between the Ct-value and the log(DNA amount) over 5 orders of magnitude (50 fg to 0.5 ng, Fig. 3a) with nearly identical slopes close to the optimal value of -3.32 for the CteA2 and the Eub primer pairs. This indicates that both primer pairs are suitable for use in qPCR analysis. A comparison of the *C. testosteroni *fractional numbers obtained by qPCR and FISH (Fig. 3b) shows identical trends for the abundances of *C. testosteroni *in the activated sludge and the following two enrichment steps. The initial *C. testosteroni *abundance was low (0.003% by qPCR, not detectable by FISH), and increased via a moderate level in the first enrichment culture (3% by qPCR and FISH) to a major proportion of the population detected by the EUB primers or probe mix (50% by qPCR and 70% by FISH) in the second enrichment culture.

### Determination of *C. testosteroni *abundance in a reactor bioaugmentation experiment via quantitative PCR

A further test of the qPCR primers CteA2 in mixed culture experiments was done on DNA samples extracted from 3 laboratory scale biofilm reactors in an experiment to introduce degradation of 3-chloroaniline (3CA) via conjugal transfer of the 3CA-degradative plasmid pNB2. A full description of the experiment can be found elsewhere [23]. Briefly, all reactors where initially inoculated with an activated sludge-derived mixed bacterial culture. Reactor PB then received a *Pseudomonas putida *strain and reactor P received a *Comamonas testosteroni *strain, both carrying plasmid pNB2, whereas control reactor N did not receive additional bacteria. The *P. putida *strain was not able to degrade 3CA, whereas the *C. testosteroni *strain was. All reactors received a feed containing 3CA and four easily degradable carbon sources (glucose, gluconate, acetate, citrate). Reactor PB started to degrade 3CA after an initial lag phase, and finally, a number of *C. testosteroni *pNB2-transconjugants capable of 3CA-degradation could be isolated from this reactor. Reactor P showed 3CA-degradation from the beginning of the experiment on, whereas no degradation could be observed in reactor N.

**Figure 4:**
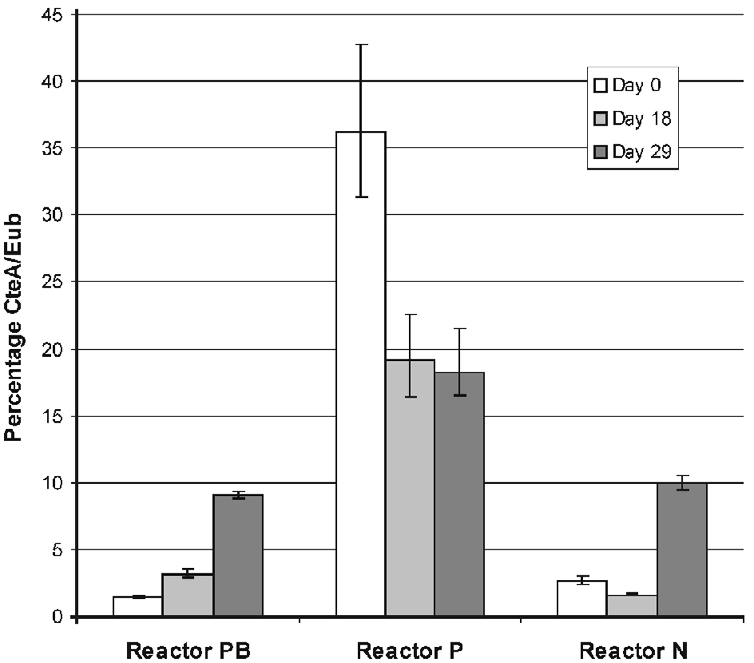
Abundances of *C. testosteroni *during a bioaugmentation experiment [23] using laboratory scale biofilm reactors inoculated with activated sludge bacteria. Reactors PB and P additionally received a *P. putida *and a *C. testosteroni *strain, respectively.

Using quantitative PCR, we determined the *C. testosteroni *abundance in DNA samples obtained at the start of the experiment (day 0) as well as on day 18 after onset of degradation of reactor P and towards the end of the experiment on day 29 (Fig. 4). Whereas initial abundance of *C. testosteroni *in reactor PB was low around 1% and rose via 3% to a final level of 9%, reactor P initially showed a high *C. testosteroni *abundance of 36% due to addition of the pNB2-carrying *C. testosteroni *strain which then dropped to values around 18%. Surprisingly, reactor N showed the same trend as reactor PB with an increase of the *C. testosteroni *abundance from low levels of 2% to finally 10%. Although the increase in *C. testosteroni *abundance in reactor PB is partly explained by growth of a 3CA-degrading, pNB2-carrying *C. testosteroni *subpopulation, this indicates that also non-degrading *C. testosteroni *strains might benefit from the general growth conditions in our reactors.

## Discussion

In our study, we present two sets of PCR primers and an rRNA directed oligonucleotide probe for use in FISH which can be applied for detection and quantification of a *Comamonas testosteroni *subgroup in mixed bacterial populations.

Relative quantifications (target group vs. a measure of whole eubacterial cell abundance) conducted by real-time PCR and FISH followed by image analysis demonstrate the same development of *C. testosteroni *populations during an enrichment process using testosterone as a carbon source. However, since neither of the two methods measure cell number, but rRNA gene copy number (PCR) and cell area (FISH), a direct numerical comparison is not possible here. Both quantification methods may display a sample-inherent bias, when the average 16S rRNA gene copy number per cell differs from the number of 16S rRNA gene copies per *C. testosteroni *cell in PCR or the average cell area in a sample differs from the average area of a *C. testosteroni *cell in FISH. Additionally, we applied the real-time PCR approach presented here to successfully determine abundance of *C. testosteroni *in a set of laboratory-scale wastewater treatment reactors inoculated with activated sludge bacteria and additionally bioaugmented with different bacteria of which one was a *C. testosteroni *strain.

Comparison of the target groups of the new probe set with the previously described probes targeting *Comamonas *species reveals that our oligonucleotides exclusively match sequences of *Comamonas testosteroni *strains. Primer CteA2-rev is somewhat less specific, but the combination with primer CteA2-for ensures specificity of the primer pair. In contrast, probe CTE matches with most sequences of the genera *Comamonas, Acidovorax *and *Hydrogenophaga*, but has mismatches with the sequences of *C. koreensis, Comamonas *sp. 12022, *C. testosteroni *SMCC B329 (clade B), and of the *Delftia *species among the sequences shown in Fig. 1. Probe PPT targets mainly *Comamonas *and *Acidovorax*, but both PPT and CTE also target a range of sequences from other genera of the *Comamonadaceae *[24]. Among the previously published probes, COM1424 has the narrowest range targeting predominantly *Comamonas *and *Delftia *[24].

## Conclusion

The oligonucleotides presented here are a useful tool to study *C. testosteroni *population dynamics in mixed microbial communities, when considering the method-inherent bias disussed above.

## Methods

### Bacterial strains and culture conditions

The following strains of *Comamonas testosteroni *were used: DSM 38, 1455, 1622, 6781, 11414, 12678, 50241, 50242, 50244, LMG 19554, SB3 [25] and SB4 [25]. In addition, *C. aquatica *ATCC 11330, *C. nitrativorans *DSM 13191, *C. koreensis *TK17 [26], *C. terrigena *TK41 [26], *Delftia acidovorans *ATCC 15668, *Delftia tsuruhatensis *SB5 [25], *Pseudomonas putida *DSM 291, *Pseudomonas aeruginosa *PAO1 [27], *Novosphingobium capsulatum *DSM 30196, *Aeromonas hydrophila *DSM 30187, *Escherichia coli *DH5α, *Serratia ficaria *ATCC 33105, *Acinetobacter calcoaceticus *BD413 [28], *Ralstonia eutropha *DSM 531, and *Burkholderia cepacia *H111 [29] were included as non-target organisms. All cultures were maintained on nutrient broth at 30°C.

### Enrichment of *Comamonas testosteroni *from activated sludge

Activated sludge was obtained from the aeration basin of the municipal wastewater treatment plant Garching (Germany). A sample representing approximately 1 mg of dry weight was inoculated into 25 ml of MMO medium [30] containing 0.05% testosterone (added as a 10% solution in DMSO) as the sole carbon source and incubated for 4 d (30°C, 150 rpm), until the disappearance of the testosterone crystals indicated degradation of the compound. 250 μl of this culture was inoculated into 25 ml of fresh medium and again incubated for 2 d, and a sample was spread onto MMO plates with testosterone which were incubated for 7 d at 30°C.

### Primer and probe design and evaluation

A multiple sequence alignment and calculation of a phylogenetic tree of a selection of 16S rRNA gene sequences of the genus *Comamonas *was done using ClustalX 1.81 [31] (see Fig. 1 for the sequences included in the analysis). Sequence stretches showing identities within *C. testosteroni *clade A sequences, but having mismatches to the remaining sequences, were used to design species-specific oligonucleotides. The sequences of the oligonucleotides which were finally used in this study are shown in Table 1.

The specificities of the primers and probes developed in this study as well as those of the probes COM1424, CTE, and PPT were checked using BLAST within the NCBI website [32] as well as the ProbeMatch tool in the RDP-II [33].

### PCR

For PCR from pure cultures, cells suspended in water were used as template DNA. DNA from activated sludge and the enrichment cultures was isolated as described previously [34].

Conventional PCR was carried out using a Mastercycler gradient (Eppendorf, Hamburg, Germany) and Qiagen HotStarTay DNA Polymerase (Qiagen, Hilden, Germany). A single reaction contained 0.2 μM of each primer CteA1-for and CteA1-rev, 0.2 mM of each dNTP, 1.5 mM MgCl_2_, 0.02 U/μl of Taq Polymerase in a volume of 20 μl. Either 1 μl of cell suspension or 10 ng of DNA was added as template DNA. The PCR protocol consisted of an initial denaturation of 15 min at 95°C followed by 30 cycles of 30 sec at 95°C, 1 min at 58°C and 2 min at 72°C and was concluded by a final extension of 8 min at 72°C. To exclude the possibility of PCR inhibition, parallel positive control reactions using the general eubacterial primers 341F and 534R were conducted for each template. The 16S rRNA genes of isolates were partially amplified using primers 27F and 1492R [35] and custom sequenced using primer 341F by MWG Biotech (Ebersberg, Germany). PCR products were analysed on 2% TAE agarose gels.

Real-time PCR was carried out in an ABI GeneAmp 5700 device (Applied Biosystems, Foster City, USA) using the Qiagen Quantitect SYBR Green PCR Kit. Each reaction was performed in triplicate and contained 12.5 μl of PCR-Mastermix, 0.2 μM of either each primer CteA2-for and CteA2-rev or 341f and 534r, 5 mM MgCl_2_, and 5 μl of template DNA in a final volume of 25 μl. The thermal protocol consisted of 15 min at 95°C followed by 40 cycles of 15 sec at 95°C and 60 sec at 60°C. The same serial 10fold dilutions of genomic DNA of *Comamonas testosteroni *DSM 50244^T ^were used to generate standard curves for both primer pairs.

### FISH and image analysis

FISH was performed as described [36], using paraformaldehyde as a fixative. For elucidation of the hybridization conditions for the probe CteA, hybridization temperature was kept constant at 46°C. The formamide concentration in the hybridization buffer was varied by 10% increments. *C. testosteroni *LMG 19554 and *C. aquatica *ATCC 11330 were used as positive and negative controls, respectively. The formamide concentration which resulted in clear strong signals for *C. testosteroni *and no signals for *C. aquatica *was used in subsequent hybridizations. Probe CteA was labelled with the cyanine dye CY3, whereas the EUB probe mix (an equimolar mixture of probes EUB338, EUB338-II, and EUB338-III) was labelled with the cyanine dye CY5. All hybridizations were performed as simultaneous dual colour hybridizations where the EUB mix served the role of a general counter stain for total bacterial cell number. Images were recorded using a Zeiss LSM 510 Meta (Carl Zeiss, Jena, Germany) confocal laser scanning microscope. For quantitative analyses, the percentages of area coverage of signals from the CteA probe and the EUB mix were calculated using the Quantimet Q500W (Leica, Cambridge, England) image analysis system. The abundance of *C. testosteroni *was calculated as the ratio of the area covered by biomass stained simultaneously with both CteA and EUB probes to the area covered by EUB-stained biomass only. For each sample, 20 microscopic fields (92 × 92 μm) were analysed.

### Accession numbers

The 16S rRNA sequences determined in this study have been deposited in the EMBL nucleotide sequence database [37] under the accession numbers [EMBL:AM113739–AM113745].

## Authors' contributions

Both authors contributed to experimental design of the project. SB carried out the experimental work and drafted the manuscript. MH revised and finalized the manuscript. All authors read and approved the final manuscript.

## Figures and Tables

**Table 1:** PCR primers and FISH probes used in this study

Name	Sequence 5'-3'	T_M_/FA^a^	Position^b^	Reference
CteA1-for	CGAAAAGCCTGGGGCTAATAT	58°C	449-469	this study
CteA1-rev	CCATCTCTGGTAAGTTCCTGC	60°C	1019-999	this study
CteA2-for	TTGACATGGCAGGAACTTACC	58°C	991-1011	this study
CteA2-rev	TCCCATTAGAGTGCTCAACTG	58°C	1157-1136	this study
341F	CCTACGGGAGGCAGCAG	60°C	341-356	[14]
534R	ATTACCGCGGCTGCTGGC	60°C	534-517	[14]
27F	AGAGTTTGATCATGGCTCAG	55°C	8-27	[35]
1492R	GGTTACCTTGTTACGACTT	53°C	1510-1492	[35]
CteA	CATGACCCGGGGATATTAGC	30%	481-462	this study
Eub338	GCTGCCTCCCGTAGGAGT	30%	355-338	[38]
Eub338-II	GCAGCCACCCGTAGGTGT	30%	355-338	[39]
Eub338-III	GCTGCCACCCGTAGGTGT	30%	355-338	[39]

^a ^Melting temperature for PCR primers or formamide concentrations for FISH probes, respectively

^b ^Position on the *E. coli *16S rRNA sequence
